# Proteome Profiling of *Mycobacterium tuberculosis* Cells Exposed to Nitrosative Stress

**DOI:** 10.1021/acsomega.1c05923

**Published:** 2022-01-14

**Authors:** Alemayehu Godana Birhanu, Marta Gómez-Muñoz, Shewit Kalayou, Tahira Riaz, Timo Lutter, Solomon Abebe Yimer, Markos Abebe, Tone Tønjum

**Affiliations:** † Department of Microbiology, University of Oslo, P.O. Box 4950, Nydalen, NO-0424 Oslo, Norway; ‡ Institute of Biotechnology, Addis Ababa University, P.O. Box 1176, Addis Ababa, Ethiopia; § Department of Microbiology, Oslo University Hospital, P.O. Box 4950, Nydalen, NO-0424 Oslo, Norway; ∥ International Center of Insect Physiology and Ecology (ICIPE), P.O. Box 30772-00100 Nairobi, Kenya; ⊥ Coalition for Epidemic Preparedness Innovations (CEPI), P.O. Box 123, Torshov, 0412 Oslo, Norway; # Armauer Hansen Research Institute, Jimma Road, P.O. Box 1005 Addis Ababa, Ethiopia

## Abstract

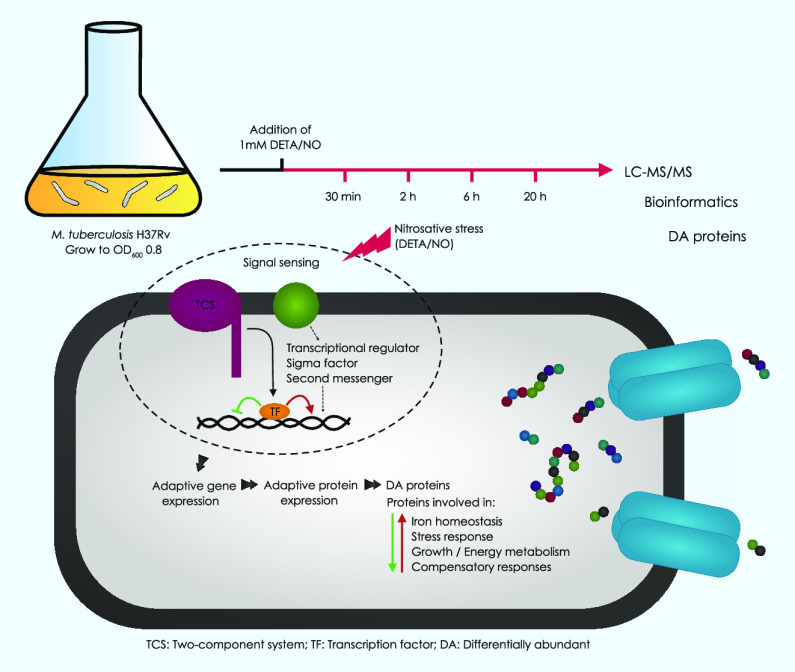

Reactive nitrogen species (RNS) are secreted by human cells in response to infection by *Mycobacterium tuberculosis* (Mtb). Although RNS can kill Mtb under some circumstances, Mtb can adapt and survive in the presence of RNS by a process that involves modulation of gene expression. Previous studies focused primarily on stress-related changes in the Mtb transcriptome. This study unveils changes in the Mtb proteome in response to a sub-lethal dose of nitric oxide (NO) over several hours of exposure. Proteins were identified using liquid chromatography coupled with electrospray ionization mass spectrometry (LC–MS/MS). A total of 2911 Mtb proteins were identified, of which 581 were differentially abundant (DA) after exposure to NO in at least one of the four time points (30 min, 2 h, 6 h, and 20 h). The proteomic response to NO was marked by two phases, with few DA proteins in the early phase and a multitude of DA proteins in the later phase. The efflux pump Rv1687 stood out as being the only protein more abundant at all the time points and might play a role in the early protection of Mtb against nitrosative stress. These changes appeared to be compensatory in nature, contributing to iron homeostasis, energy metabolism, and other stress responses. This study thereby provides new insights into the response of Mtb to NO at the level of proteomics.

## 1 Introduction

*Mycobacterium tuberculosis* (Mtb) is a successful human pathogen, with the ability to survive the effects of host–defense mechanisms, including intracellular and secreted antibacterial reactive nitrogen and oxygen species (RNS/ROS) produced by macrophages. Mtb-infected macrophages generate nitric oxide (NO) and other RNS via the inducible nitric oxide synthase (iNOS).^1^ Although RNS can be lethal, Mtb is able to protect itself against the devastating effect of RNS by direct scavenging, iron sequestration, suppression of RNS production, catalytic detoxification, and other stress responses, including NO-inducible DosR-dependent repair of RNS-mediated cellular damage.^2^

In response to host-generated RNS/ROS, Mtb upregulates expression of redox-scavenging enzymes, including catalase (KatG), superoxide dismutases (SodA and SodC), and the peroxynitrite reductase and peroxidase complex (PNR-P) encoded by *ahpC*, *ahpD*, *lpd*, and *sucB* (*dlaT*).^2a,3^ In analogy, mice deficient in *dlaT* expression are hyper-susceptible to RNS.^3^ Another defense against RNS involves oxygen-dependent catalytic detoxification of NO, which is mediated by truncated hemoglobin (trHbN) and prevents inhibition of aerobic respiration by NO. Other studies have shown that Mtb strains carrying mutations in the gene encoding methionine sulfoxide reductase (msrA), the Mtb proteasome (prcBA), nucleotide excision repair (uvrB), or F-420 biosynthesis (fbiC), are hyper-susceptible to RNS,^2a^ suggesting that these proteins protect Mtb against the potentially lethal effects of RNS. Exposure to NO-induced stress upregulates expression of alpha crystalline (Acr), bacterioferritin (BfrB), and the DosR regulon in Mtb; however, little is known about the role of these Mtb genes/proteins in the response to nitrosative stress.^2a^

Previous studies revealed high levels of iNOS in tuberculous lesions from the lung and in macrophages in bronchoalveolar lavage from patients with tuberculosis.^4^ The high level of iNOS in human alveolar macrophages is critical for their microbicidal activity^2b^ and could contribute to the clearance of Mtb in human granulomas. This is consistent with the bacteriostatic and bactericidal activity of RNS *in vitro*,^2a^ the Mtb susceptibility of mice deficient in NOS_2_,^5^ and the suppression of Mtb-killing activity of human macrophages by inhibitors of iNOS.^2a,4^ NO reacts extensively with multiple macromolecules and induces a systems-level stress response;^6^ therefore, investigating the proteome of NO-treated Mtb cells provides an opportunity for a detailed analysis of the global proteomic response to nitrosative stress.

Following uptake by macrophages, the exposure of Mtb to RNS represents a major component of the host cell immune response.^7^ Furthermore, intracellular release of NO is also an important anti-Mtb mechanism of a class of nitroimidazole drugs used in the treatment of tuberculosis.^8^ NO can reversibly inhibit aerobic respiration and growth in Mtb.^9^ It also modulates a regulon comprising 48 genes, known as the “Mtb dormancy regulon”, which mediates physiological transition and adaptation to dormancy.^9^ Furthermore, proteins related to house-keeping, energy metabolism, DNA repair, and iron acquisition are altered by exposure to NO stress.^10^ Nitrosative stress can also promote degradation of proteins containing iron–sulfur [Fe–S] clusters.^11^

The mechanisms and/or pathways that protect Mtb against host defense-related antimicrobials could play roles in the process leading to bacterial persistence; therefore, better understanding of these mechanisms could facilitate discovery of new tools to treat or cure persistent Mtb-associated infection and/or pathology.^2a^ Many pathogens, including Mtb, must detoxify NO and repair RNS/ROS-damaged biomolecules before the pathogen can establish a productive infection.^6^ Agents that inhibit a pathogen’s defense system may have the potential to be used as “next-generation” antibiotics.^6^ In order for this approach to succeed, more information about the pathogen-host interactions is needed. For example, it would be useful to document and analyze changes in the Mtb transcriptome, proteome, and metabolome and the occupancy of transcription factor binding sites in response to various stresses, including altered redox environment.^12^

The aim of the present study was to investigate changes in the Mtb proteome in response to nitrosative stress, including the kinetics of this response over time. Building on previously published data, including our own, we used an experimental model in which Mtb cells are exposed to a sub-lethal dose of diethylenetriamine/nitric oxide adduct (DETA/NO), which is used here as the NO donor.^2a,10c,11^ Exposure of Mtb cells to DETA/NO *in vitro* mimics the major microbicidal activity experienced by Mtb in TB infections. We have previously monitored the global Mtb response to DETA/NO at the transcriptional level.^10c^ Young and co-workers compared DETA/NO-treated Mtb cells at the transcriptional and proteomic levels.^11^ Here, more in-depth proteomic analyses of DETA/NO-treated Mtb cells were performed at four time points over 20 h. Bioinformatics analyses of the proteome data provided new insights into the kinetics of the Mtb response to nitrosative stress. This study enlightens the response of Mtb to NO, which is crucial to devise a control strategy against the deadly human pathogen Mtb.

## 2 Results

### 2.1 Differentially Abundant Proteins in Mtb Cells Exposed to Nitrosative Stress

This study analyzes the global changes in the proteome of Mtb cells in response to nitrosative stress induced by sub-lethal doses of DETA/NO over time. We identified 33,509 peptides assigned to 2,911 proteins, which accounts for 72.9% of the predicted Mtb proteome (Table S1). This is in line with previously published studies on the Mtb proteome.^13^ A total of 186 and 36 of these proteins were exclusively detected in stressed and control samples, respectively (Table S2). Two thousand four hundred ninety proteins were represented and exhibited valid LFQ values in at least 50% of the samples; only these data points were subject to the quantitative proteomic analysis (Table S3). The two-sample *t*-test was employed to define the presence of the differentially abundant proteins at 30 min, 2 h, 6 h, and 20 h after exposing the Mtb cells to NO (Table S4).

**Figure 1:**
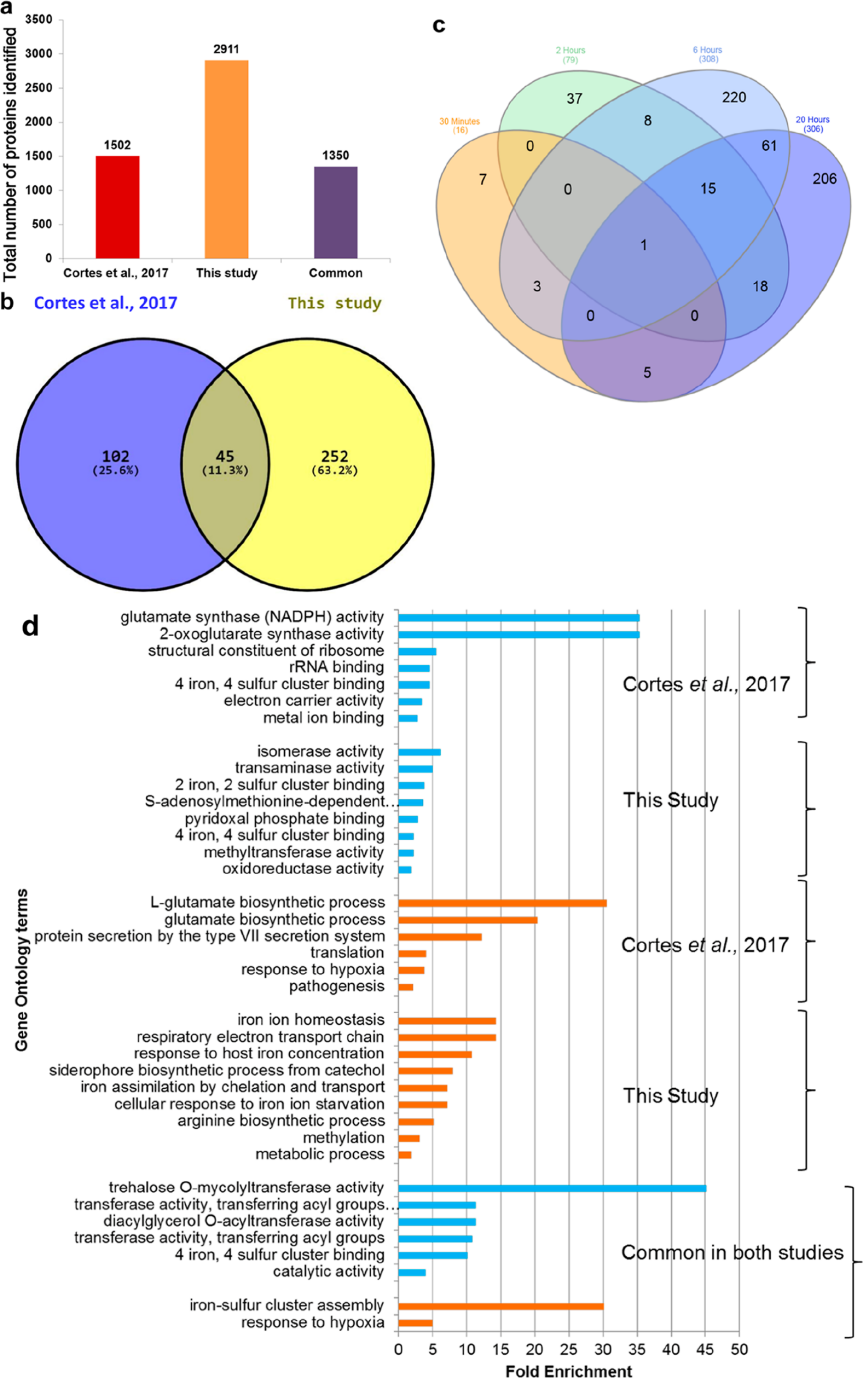
Protein identification and quantification in Mtb exposed to nitrosative stress. The abundance of proteins identified by Cortes et al., 2017 vs this study (a). The Venn diagram of DA proteins identified by Cortes et al., 2017^11^ vs this study (b). The Venn diagram of DA proteins identified by this study at four time points (c), which illustrates the number of DA proteins in Mtb treated with 1mM DETA/NO for 30 min (orange), 2 h (green), 6 h (light blue), and 20 h (deep blue). The gene ontology analysis of DA proteins identified by Cortes et al., 2017^11^ vs this study represented by blue bars (molecular function) and orange bars (biological processes) (d).

**Figure 2:**
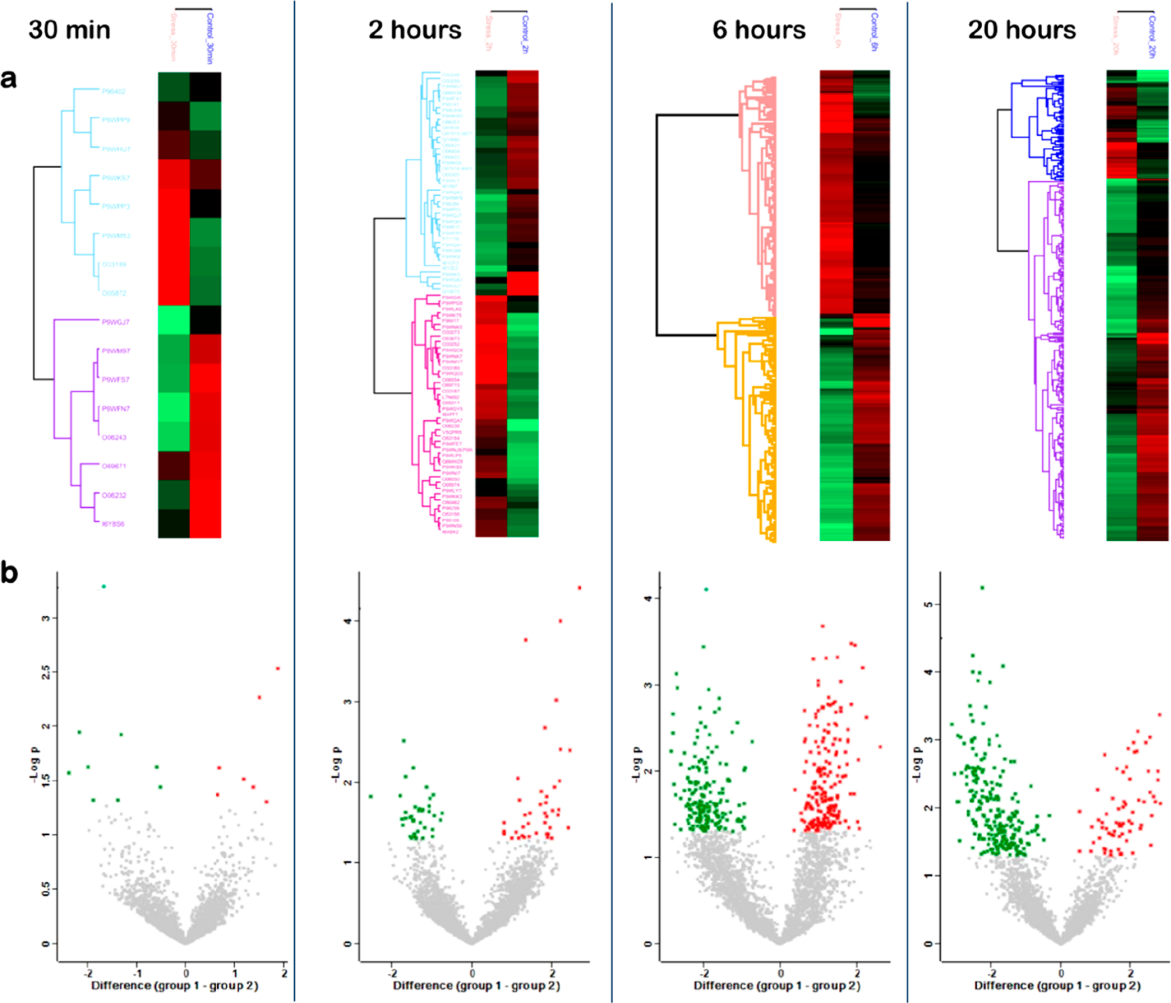
Quantitative proteomic analysis after exposure to nitrosative stress for each time point. (a) Heat maps of DA proteins after 30 min, 2 h, 6 h, and 20 h exposure to DETA/NO versus the control. The color code indicates the abundance of each protein from bright red (more abundant) to light green (less abundant). (b) Volcano plots of log2 fold changes for DA proteins after 30 min (**7 up**, **9 down**), 2 h (**41 up**, **38 down**), 6 h (**193 up**, **182 down**), and 20 h (**69 up**, **237 down**) exposure to DETA/NO versus the control. Red dots correspond to upregulated proteins and green dots to downregulated proteins. The list of DA protein in each time point is provided in Table S4a–d.

The number of differentially abundant (DA) Mtb proteins at each time point is also shown in Figures 1c, 2a,b, and Table S4a–d. The number of DA proteins was significantly higher at 6 h (*n* = 375) and 20 h (*n* = 306) than at 30 min (*n* = 16) and 2 h (*n* = 79) (Table S4, Figure 1). All these DA proteins were further analyzed through hierarchical clustering, and the results were visualized as heat maps and volcano plots (Figure 2). The lists of DA proteins in Mtb exposed to nitrosative stress for the different time points can be found in the Supporting Information (Table S4a–d). Additionally, the names of the DA proteins that are common in at least two-time points are given in Table S5. The findings in this study were compared with the study by Cortes et al., 2017^11^ and Namouchi et al., 2016^10c^ (Table S8). Our study has identified a much higher number of proteins than in the study by Cortes et al., 2017 (Figure 1a,b). The gene ontology analysis of DA proteins in both studies revealed modulation of similar biological functions and biological processes (Figure 1d).

Rv1687c and Rv1405c were among the proteins that were most predominantly DA after nitrosative stress. The predicted efflux pump Rv1687c was the only protein to be more abundant at all the time points after NO stress. A proposed 3D structure/high-quality homology model of Rv1687c was retrieved from SWISS-MODEL based on *Escherichia coli* LptB-E163Q in complex with ATP-sodium; https://swissmodel.expasy.org/repository/uniprot/O33189.

**Figure 3:**
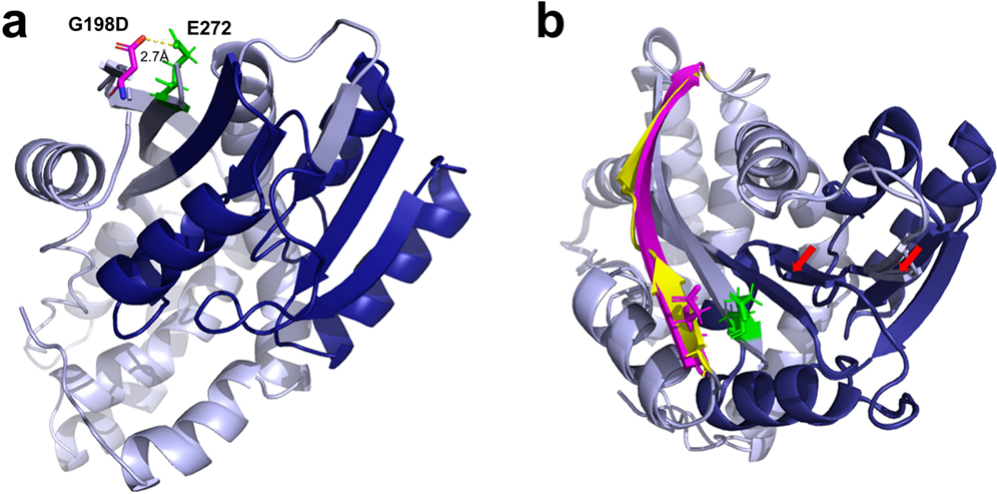
Predicted 3D structure of the proposed methyltransferase Mtb Rv1405c (a). The initial prediction of the tertiary structure of Mtb Rv1405c using I-TASSER revealed that the closest structure available for homology modeling is the crystal structure of the S-methyltransferase TmtA from *A. fumigatus* Z5 (PDB: 5EGP). A C-score of 0.09 was calculated for this predicted structure. Light blue = predicted Rv1405c G198D structure, dark blue = putative methyltransferase domain, purple = G198D mutation, and green = E272 as part of the opposite β-strand. Predicted structures of Rv1405c wt and G198D were superimposed (b). G198 is part of a short β-strand (yellow) which is not part of but close to the methyltransferase domain, while G198D induces a 90° rotation, resulting in a prolonged β-strand (purple). Predicted structural changes in the putative methyltransferase domain are indicated by red arrows.

The initial prediction of the tertiary structure of Mtb Rv1405c using I-TASSER^14^ revealed that the closest structure available for homology modeling was the crystal structure of the S-methyltransferase TmtA from *Aspergillus fumigatus* Z5 (PDB: 5EGP). A C-score of 0.09 was calculated for the predicted structure for the proposed methyltransferase 1405c, as shown in Figure 3.

The genes encoding Rv1687c and Rv1405c in antibiotic-sensitive and antibiotic-resistant Mtb strains through evolution were highly conserved (Figures S1 and S2). With very few exceptions, single nucleotide polymorphisms (SNPs) had no effect on the amino acid sequence. For Rv1687c, there was one aspartic acid to asparagine mutation in *Mycobacterium africanum*, as well as one silent mutation. For Rv1405c, three of the four SNPs were silent mutations, where only one SNP in Mtb lineage 1 conferred an amino acid change of glycine to aspartic acid (G198D). The consequence of the SNP in Mtb L1 HN-024 Rv1405 is a G198D mutation, and so, there will be an Asp sitting in the β-strand (negative charge). In the opposite β-strand (green), there is a glutamic acid (negative charge). Pfam predicts a methyltransferase domain at position 54–149. Gly198 is part of a β-strand in close proximity to the methyltransferase domain. The G198D mutation is very likely to have an impact on the protein structure, as the resulting aspartic acid is sitting opposite to Glu272 of the neighboring, antiparallel β-strand (Figure 3). A root-mean-square deviation of 0.724 was calculated for superimposed wt and G198D structures. The G198D mutation seems to tilt the β-strand by an angle of 90°. Furthermore, I-TASSER predicts a prolonged β-strand (197–209) for G198D, as opposed to the wild-type structure, where two smaller β-strands are located 197–201 and 204–209. Minor structural changes in the putative methyltransferase domain are predicted (Figure 3).

In summary, both genes were highly conserved, which points to their functional significance. Both the Rv1687c and Rv1405c genes are located in the reverse complement orientation in the Mtb H37Rv genome. However, in some nontuberculous mycobacterial species, the Rv1687c and Rv1405c genes were not reverse-complement, but on the opposite strand. In *Mycobacterium avium*, *Mycobacterium indicus*, *Mycobacterium intracellulare*, *Mycobacterium liflandii*, *Mycobacterium smegmatis,* and *Mycobacterium vanbaalenii*, Rv1687c was on the opposite strand, while in *M. avium*, *M. indicus*, *M. intracellulare,* and *M. liflandii*, Rv1405 was on the opposite strand.

### 2.2 Two-Phased Response of Mtb Cells Exposed to Nitrosative Stress

**Figure 4:**
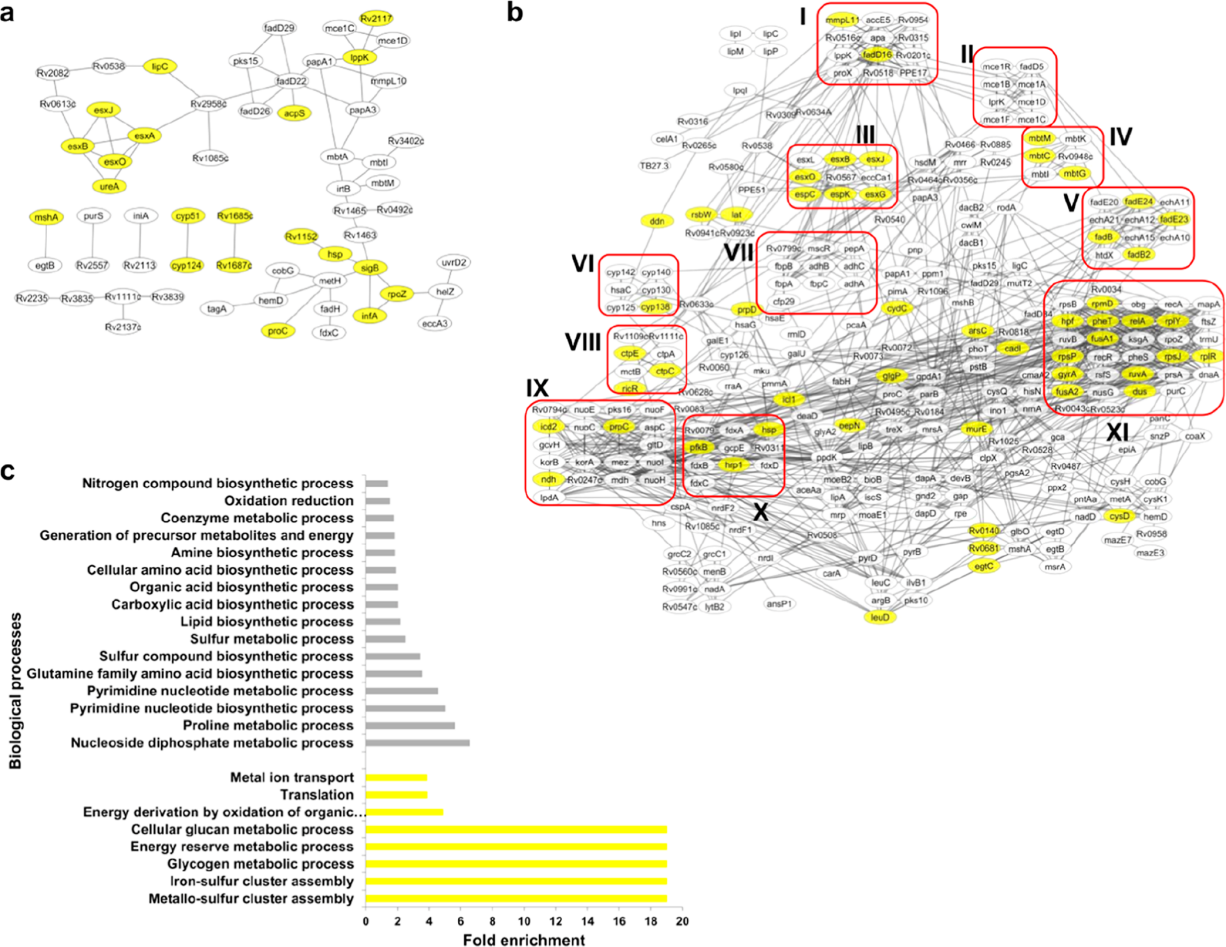
Protein interaction network and gene ontology analysis of early and late DA proteins. The protein interaction networks were generated separately for the early responsive DA proteins (30 min and 2 h) (a) and proteins altered at the later time points (6 h and 20 h) (b). Biologically important pathways are indicated from **I** to **XI**. These clusters are grouped based on their role in Mtb physiology. **I and V**: fatty acid metabolism, **II**: mammalian cell entry proteins, **III**: type VII secretion system proteins, **IV**: acyl carrier proteins, polyketide synthesis, and monooxygenase, **VI**: cytochromes, **VII**: alcohol dehydrogenases and antigen 85 families, **VIII**: metal cation transporter ATPase, **IX**: NADH dehydrogenases, **X**: ferredoxins and stress response proteins, and **XI**: proteins involved in information pathways. The gene ontology analysis was performed for the DA proteins at the later time points (c). **Yellow**: highly abundant and **gray**: less abundant. The Benjamini–Hochberg and Bonferroni adjusted values for each GO term are shown in Table S7a,b.

As described in Table S4, only few changes in protein abundance occurred during the early stages (30 min and 2 h) of exposure to nitrosative stress, while several changes were observed in the later stages (6 and 20 h). The protein interaction networks were generated using the DA proteins at the two phases (early and late) separately. In the early phase after exposure to nitrosative stress, a number of the 6 kDa early secretory antigenic target ESAT-6-like proteins (EsxA, EsxB, EsxJ, and EsxO), Hsp and proteins involved in information pathways (InfA, RpoZ, and SigB) were more abundant (Figure 4a). In the later phase of exposure to nitrosative stress, the abundance of many proteins (circled in red lines) involved in several pathways was affected (Figure 4b). These includes ESAT-6-like proteins, proteins involved in lipid metabolism, respiration and information pathways, metal cation transporter ATPase (CtpA, CtpC, and CtpE), ferredoxin (FdxA, B, C, and D), secreted antigen 85 families (FbpA, FbpB, and FbpC), alcohol dehydrogenases (AdhA, AdhB, and AdhC), mammalian cell entry proteins, and proteins involved in the siderophore biosynthetic process (MbtC, MbtG, MbtI, MbtK, and MbtM). Proteins in yellow are more abundant, while those in gray are less abundant after exposure to nitrosative stress. Furthermore, the gene ontology of DA proteins in the later phase was depicted. This analysis revealed a number of biological processes to be affected by NO stress (Figure 4c), including iron homeostasis, iron–sulfur cluster assembly, and a number of other metabolic processes.

### 2.3 Clusters of DA Mtb Proteins Affected by Nitrosative Stress

**Figure 5:**
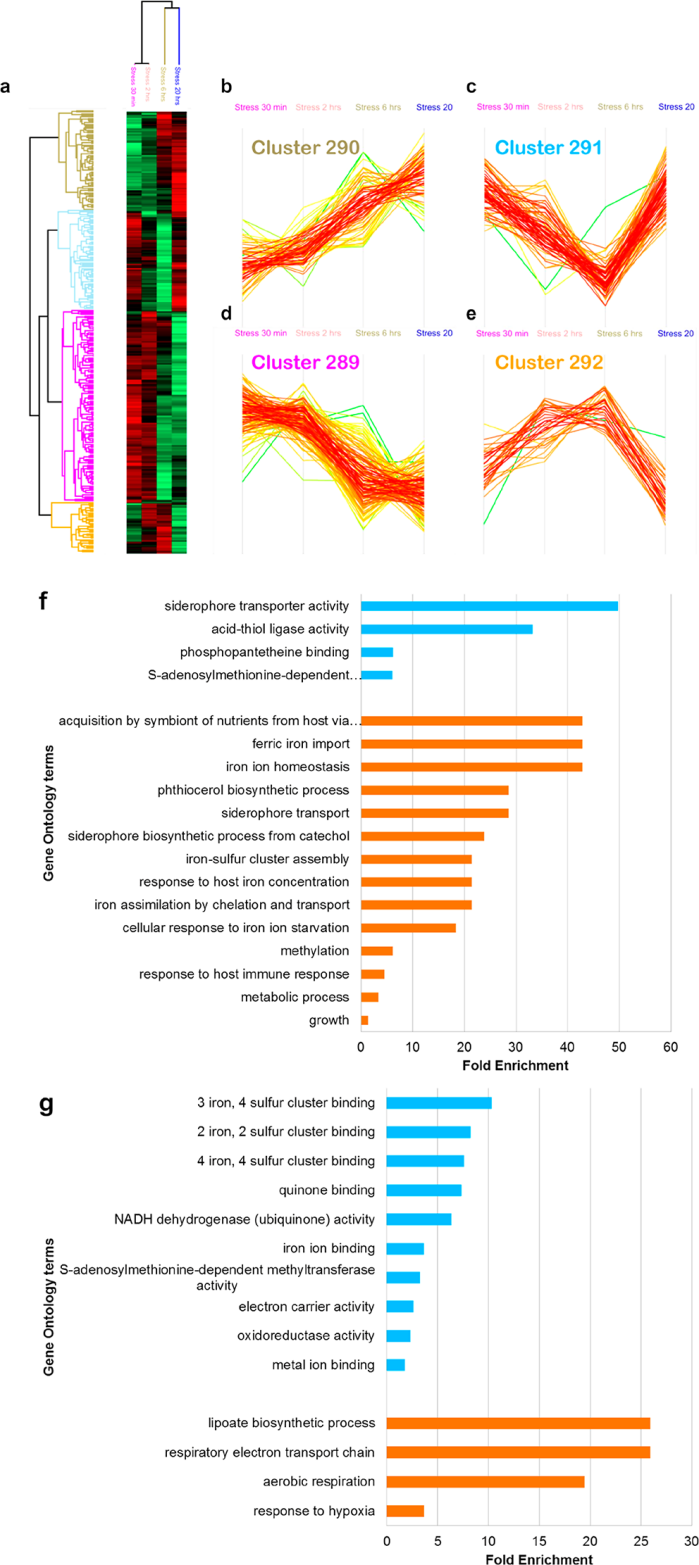
Quantitative proteomic analysis after exposure to nitrosative stress over time. The heat-map reflects the changes in abundancy of certain proteins over 20 h. The color code indicates the abundancy of each protein from bright red (more abundant) to light green (less abundant) (a). This clustergram represents the profiles of NO stress-induced DA proteins (*n* = 297), which fall into 4 separate clusters; 290 (upregulated over time) (b), 291 (downregulated after 30 min and 2 h and then upregulated after 6 and 20 h) (c), 289 (downregulated over time) (d), and 292 (upregulated after 30 min and 2 h and then downregulated after 6 and 20 h) (e). The color of the cluster names corresponds to the four respective members in the heatmap. The gene ontology analysis of proteins in cluster 290 (f) and cluster 289 (g) is represented in **blue** (molecular function) and **orange** (biological process), respectively.

The changes in Mtb protein abundances over time in response to NO were investigated using ANOVA. Two hundred ninety seven proteins were significantly DA across the four time points (Table S6). These DA proteins were subjected to hierarchical cluster analysis, and four different clusters were detected (Figure 5a). These clusters reflect the DA proteins according to their expression profile over time. The protein interaction network for each respective cluster is also presented.

The first cluster (cluster 290) comprised proteins whose expression increased over time. It included proteins involved in iron ion acquisition and repair of iron–sulfur cluster proteins such as ferric iron importers (IrtA and IrtB), proteins involved in mycobactin biosynthesis (MbtA, MbtC-G, and MbtI-N), the type VII secretion system proteins (ESX-3) (EccA3, EccD3, EccE3, and EspG3), and the sulfur formation (SUF) operon (Rv1461-Rv1463, Rv1465, and Rv1466). It also included proteins involved in detoxification (e.g., KatG and IniA) and in carbon metabolism (e.g., Icl1) (Figure 5b, Table S6).

The second cluster (cluster 291) represents proteins that were downregulated during the first 6 h after the addition of DETA/NO, but were then upregulated in the last 14 h. In this cluster, proteins involved in arginine biosynthesis (ArgB, ArgC, and ArgD) are found (Figure 5c, Table S6).

The third cluster (cluster 289) includes proteins whose expression decreased along 20 h of exposure to DETA/NO (Figure 5d, Table S6). Proteins in this cluster include ferredoxins (FdxA, FdxC, and FdxD) and subunits of the NADH-quinone oxidoreductase (NuoC, NuoE, NuoF, and NuoI).

The fourth cluster (cluster 292) includes proteins that were upregulated during the first 6 h after exposure to DETA/NO, but then downregulated along the last 14 h. These proteins are involved in translation such as the 50s ribosomal proteins (RplP and RpmC) and Hsp and GatC (Figure 5e, Table S6).

As shown in the gene ontology analysis, proteins involved in iron homeostasis were upregulated in time (Figure 5f), while the expression of iron–sulfur [Fe–S] cluster proteins was downregulated (Figure 5g).

### 2.4 Proteins Involved in Respiration and Iron Homeostasis Are Induced under Nitrosative Stress in Mtb

**Figure 6:**
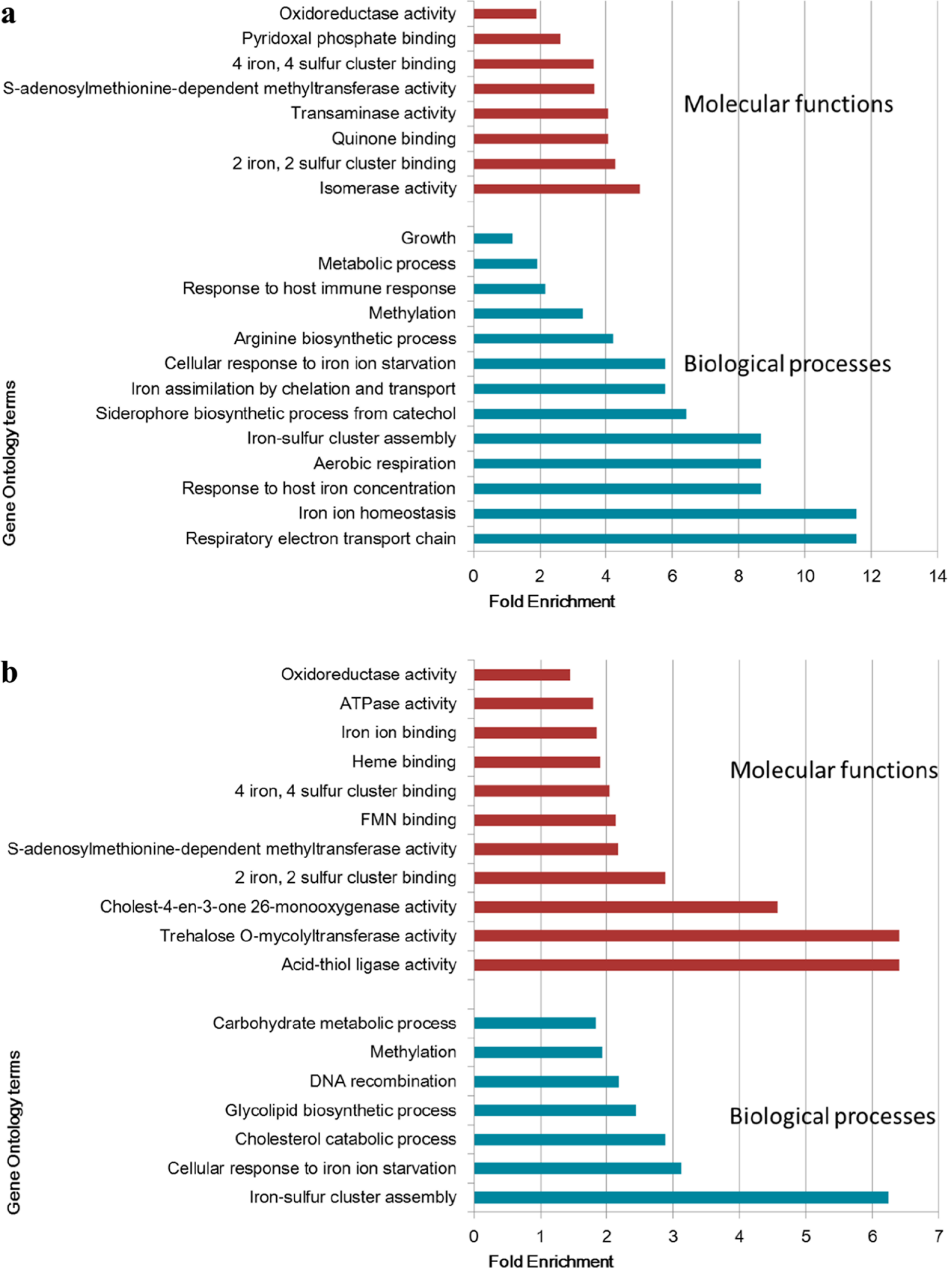
Gene ontology analysis of DA proteins in Mtb exposed to nitrosative stress. The biological process and molecular functions enriched from DA proteins over the four time points (ANOVA significant) (a) and at each time-point combined (b). The Benjamini–Hochberg and Bonferroni adjusted values for each GO term are shown in Table S7c,d.

The gene ontology analysis of DA proteins (ANOVA) showed that proteins involved in respiration and iron homeostasis were among the highly enriched biological processes and biological functions (Figure 6a). A separate gene ontology analysis was performed on the total proteins that were DA at the four time points combined (Figure 6b). The fold enrichment indicated how much proteins in a specific pathway are over-represented. The biological processes identified included carbohydrate and glycolipid metabolism, Fe–S cluster assembly, cellular response to iron starvation, and DNA recombination, and the molecular functions identified were oxidoreductase activity iron ion-/heme binding, Fe–S cluster binding, and monooxygenase activity. Furthermore, DNA/RNA metabolism and growth were enriched among Mtb proteins expressed exclusively under NO stress (Table S7e).

## 3 Discussion

Most of the former efforts to elucidate the survival strategies of Mtb against NO are based on microarray analysis^2a,9,15^ and transcriptomic studies, including our own.^10c,11^ These studies elucidate the ability of Mtb to counteract nitrosative stress based on rapid changes in gene expression at the transcriptional level.

Here, we convey the global Mtb proteome over time, consistent with a two-phased response of Mtb cells to nitrosative stress. Notably, we detected few changes in the expression in the early phase (30 min and 2 h), followed by more extensive compensatory (adaptive) responses in the later phases (6 and 20 h). This delayed response at the proteomic level confirms previously described findings by Cortes et al., 2017.^11^

### 3.1 Mtb Efflux Pumps under Nitrosative Stress May Confer Antimicrobial Resistance

Despite a predominant delayed response, Rv1687c predicted to encode an efflux pump,^16^ stood out as being the only protein consistently more abundant in Mtb cells exposed to nitrosative stress at all the four time points. This putative efflux pump might play a role in the early protection of Mtb against nitrosative stress.^17^ The (Rv1686c)_2_/(Rv1687c)_2_ ABC transporter has been classified as an antibiotic transporter with unknown function.^16^ While Rv1687c was highly abundant at all the time points investigated, Rv1685c, annotated as a transcriptional regulator from the TetR family, was also more abundant in cells treated with NO after 2, 6, and 20 h.

The putative drug membrane transporter RV1687c is known to be upregulated when mycobacteria are exposed to stressors.^18^ Rv1685c/Rv1686c/Rv1687c are also upregulated by acid-nitrosative stress^19^ and antibiotics.^20^ Transcriptomic studies on Mtb isolates from drug-compliant patients showed activation of drug efflux pumps, like Rv1687c, when comparing the susceptible isolate at the beginning of the treatment to the multidrug-resistant isolate at a later point of the treatment.^18a^ Consequently, it has been speculated that this efflux pump could be involved in resistance to antibiotics; however, overexpression or absence of the (Rv1686c)_2_/(Rv1687c)_2_ ABC transporter does not influence the resistance of Mtb mutants to a wide spectrum of antibiotics, including isoniazid.^21^ It is noted that some antibiotics, such as isoniazid, can act as NO donors.^8,22^ The role of efflux pumps in the defense against stress has also been observed in other bacteria.^23^ Taken together, the (Rv1686c)_2_/(Rv1687c)_2_ transporter might not be directly involved in antibiotic resistance, but it seems to be important as a detoxification system to reduce or cope with the cellular damage of certain antitubercular drugs and antimicrobial compounds. Hence, understanding the response of Mtb to nitrosative stress will enable us to develop improved strategies to treat tuberculosis.

### 3.2 Methylation in Mtb under Nitrosative Stress

Acid stress has been shown to upregulate the expression of the virulence-associated methyl transferase Rv1405c.^24^ Rv1405c was the second most DA protein, when exposed to 20 h of DETA/NO treatment, compared to the control. The orthologue of Rv1405c in *Mycobacterium bovis* (Mb1440c) is highly upregulated in response to *in vitro* and *in vivo* acid shock.^25^ Rv1405c is strongly induced in macrophages and has been identified as important for virulence, and therefore, the methylation of target(s) of Rv1405c could play a role in response to stress and virulence.^26^ Rv1405 is reported to promote drug refractoriness in Mtb.^27^

Some Mtb methyltransferases can alter host gene expression by methylating histone tails or host DNA,^28^ while others can modify lipids, such as the mycobacterial mycolic acids.^29^ Analysis of Mtb methyltransferases revealed that 10 of them are virulence factors.^29^ Interestingly, half of these methyltransferases associated with virulence were less abundant in our study after 6 h (Rv2959c) and 20 h [PcaA (Rv0470c), MmaA4 (Rv0642c), Rv2952, and Rv2954c]. *mmaA4*, *Rv2952*, *Rv2954c*, and *Rv2959c* are also found to be downregulated in transcriptional studies.^2a,10c,11^ Notably, the high abundancy of the methyltransferase Rv1405c, contrasting the low abundance of other methyltransferases related to virulence under nitrosative stress, might indicate a distinct role for Rv1405c under nitrosative stress. Despite its high level of conservation, we found one nonsynonymous SNP in HN-024 Rv1405c, inducing a G198D mutation (Figure 3). Situated opposite to Glu272, this amino acid’s substitution is very likely to have an impact on the 3D structure, as both Asp198 and Glu272 are negatively charged. Furthermore, the superimposed structures indicated minor changes in the neighboring putative methyltransferase domain.

### 3.3 RNS Detoxification Strategies

Mtb also counteracts nitrosative stress through detoxification mechanisms. Some of the genes encoding catalase peroxidase (*katG*), superoxide dismutase (*sodA*), alkyl peroxide reductase (*ahpCDE*), thioredoxin (*thiX* and *trxB1C*), or thioredoxin reductase (*trxB2*) have been reported to be upregulated upon nitrosative and oxidative stresses.^2a,10c,11^ However, at the protein level, most of them were not DA, with exception of TrxA and TrxB1 (more abundant after 6 h). The lack of changes in abundancy of some recognized proteins involved in resistance against nitrosative stress could be related to their high basal expression levels.^2a^

Additionally, as an alternative antioxidant defense, Mtb relies on the metabolic plasticity of its central carbon metabolism.^30^ On top of their canonical function in the central carbon metabolism, enzymes like Icl1, LpdC, DlaT, HoaS (Rv1248c), or AceE are also involved in protecting Mtb from oxidative and nitrosative stresses.^31^ Icl1 was one of the most DA proteins after 20 h of DETA/NO treatment in our study. This enzyme, besides having a dual metabolic role in the glyoxylate shunt and in the methylcitrate cycle, also facilitates drug tolerance.^31a,32^ In addition to Icl1, two of the three specific enzymes of the methylcitrate cycle (PrpC and PrpD) were also highly DA after 20 h of DETA/NO treatment. Icl1, PrpC, and PrpD are required for growth inside macrophages, but while Icl1 has an essential role *in vivo*; the absence of PrpCD have no effect on growth and persistence of Mtb in mice.^33^

LpdC and DlaT, together with AhpD and AhpC, form the NADH-dependent peroxidase and peroxynitrite reductase (PNR-P) complex to detoxify RNS.^31b^ In the absence of LpdC and NADH, the PNR-P system can also be fueled by HoaS or AceE.^31c^ Despite the importance of PNR-P systems in resistance to nitrosative stress and virulence in Mtb, most of these components of the complexes were not upregulated after DETA/NO treatment. Only the expression of AhpC was increased over the first two time points and then declined from 6 and 20 h. Some metabolites, including AhpC, which play a central role in core carbon metabolism, might be repurposed to protect the Mtb pathogen via the noncanonical pathway under nitrosative and oxidative stress rather than being induced.

A highly conserved mycobacterial protein Rv3290c is a lysine ε-aminotransferase (LAT) upregulated after 20 h in response to NO. Rv3290c plays a role in the mycobacterial persistence/latent phase and amino-acid metabolism.^34^

### 3.4 Respiration under Nitrosative Stress

NO is also known to cause reversible bacteriostasis in Mtb.^9^ Inhibition of aerobic respiration^9,35^ and downregulation of protein synthesis^2a^ can contribute to such a growth arrest. We observed a switch from a more efficient to a less efficient respiration after 20 h of exposing Mtb cells to nitrosative stress, which agrees with previous studies.^2a,9,11,35^ This switch was marked by a reduction in abundancy of 5 out of 14 subunits of the proton-pumping type I NADH dehydrogenase Nuo (NuoC, NuoE, NuoF, NuoH, and NuoI) and an increase in abundance of the nonproton-pumping type II NADH dehydrogenases Ndh and of CydC (Figure 4b, cluster IX). The changes in expression of electron transport chain components were more evident at the transcriptional level, with downregulation of 13 out of 14 genes encoding Nuo (*nuoB-N*) and upregulation of Ndh and all the components of the cytochrome *bd* complex (*cydABCD*).^10c^

### 3.5 NO and the Dormancy Regulon

Based on former transcriptomic studies, sub-lethal concentration of NO and oxygen limitation have been shown to competitively modulate the expression of the 48-gene dormancy regulon (DosR) by reversible inhibition of aerobic respiration and growth.^9^ Our analysis revealed that proteins involved in the dormancy regulon were DA in at least one of the time points. Proteins like Rv0079, Rv2004c, Rv3127, Acg, PfkB, Tgs1, and Rv2623 (Hrp1) were more abundant after 20 h of incubation with DETA/NO, while Rv1812c, Rv1998c, Rv2005c, Rv2623 Rv1998c, and FdxA were less abundant. These findings were supported by previous studies reporting NO-induced changes in gene expression in the DosR regulon in mycobacteria.^9,11,36^ It has been shown that DosR enzymes, such as triacylglyceride synthase (Tgs1), ferredoxin (FdxA), and phosphofructokinase B (PfkB), assist in adapting metabolic processes to anaerobic conditions.^36d^ Notably, the expression of the two-component regulatory system proteins Senx3 and TrcR as well as the phosphate transporter PstB were altered by exposure to NO. NO interacts with the heme protein SenX3 and modulates its kinase activity and thereby its role in Mtb metabolic adaptation, including latency and reactivation.^37^

### 3.6 Iron Homeostasis under Nitrosative Stress

Distinct effects of nitrosative stress on Mtb iron-containing enzymes were observed. Iron–sulfur (Fe–S) clusters are essential cofactors that participate in vital cellular processes such as electron transfer in respiration or gene expression regulation in adaptation to stress.^38^ NO interacts with Fe–S clusters and heme groups in proteins,^36a^ leading to a metabolic remodeling that causes degradation and repair of Fe–S proteins and a switch from aerobic to anaerobic respiration.^10c,11,36a,39^ Changes in the proteome driven by NO affect Fe–S proteins and haemoproteins, including depletion of ferredoxins (FdxA-D) and cytochrome P450 enzymes (Figure 4b, clusters X and VI). The Fe–S cluster carrier protein Mrp was also less abundant after 6 h of exposure to NO. Considering that the corresponding genes are either induced or not differentially regulated,^2a,10c^ the reduction in abundancy of these proteins might be linked to their degradation as previously mentioned.^11^

Unlike other bacteria, Mtb has only one system involved in the assembly and transport of Fe–S clusters, the SUF system.^40^ Six out of seven proteins that are encoded by the *suf* operon (Rv1461-Rv1466) were more abundant after 20 h of nitrosative stress. This result is in line with previous studies at the RNA level.^2a,10c,11^

The repair of Fe–S clusters requires iron. Mycobactins are siderophores that participate in iron acquisition.^41^ The siderophore biosynthesis is controlled by IdeR, an iron-dependent regulator. Voskuil and colleagues have already pointed out that oxidation of the iron-center of IdeR by NO can simulate iron-limiting conditions.^2a^ Besides, we found other proteins encoded by genes repressed by IdeR that were more abundant after 20 h of nitrosative stress compared to the control: EspG3 and EsxG.

EspG3 is a presumable chaperone that, together with the ATPase EccA3, forms the cytosolic apparatus of the ESX-3 type VII secretion system.^42^ ESX-3 is essential for viability and is implicated in mycobactin-mediated iron uptake^43^ and in Mtb virulence. EsxG is exported by ESX-3 and is linked to inhibition of phagosome maturation.^44^ The expression of EspG3, EccA3, and membrane proteins of the ESX-3 core channel (EccD3 and EccE3) showed an increasing trend along the 20 h of treatment (Figure 5b). A former study demonstrated that ESX-3 and its secreted substrates were upregulated under NO stress.^10c^ The ferric iron importers, IrtA and IrtB, are upregulated during iron starvation.^45^ The levels of these two proteins showed a trend of increasing expression across the 20 h of nitrosative stress.

Even though free iron can accelerate cellular imbalance by generating damaging hydroxyl radicals via the Fenton reaction,^46^ evidence shows that mycobacteria invest heavily on importing iron to meet the need to repair iron-containing proteins.

## 4 Conclusions

This study presents a quantitative proteomic analysis of mycobacterial adaptation to DETA/NO, providing an improved understanding of the dynamic response of Mtb to nitrosative stress. Bioinformatics analysis of the DETA/NO-induced Mtb proteome revealed a two-phased response, with more extensive changes in protein abundances at later time points (6 and 20 h). However, the efflux pump Rv1687c appeared to be important also as a mechanism in the early phase of the defense against nitrosative stress in Mtb. The upregulation of this efflux pump under other forms of stress, including antibiotic treatment, makes it very interesting. We also observed proteins involved in many important biological processes that were affected by DETA/NO, such as methylation, iron homeostasis, respiration, and ESX-3 secretion. Complementary studies at the level of transcriptomics, proteomics, and metabolomics as well as 3D structural biology will facilitate a more in-depth understanding on how the transcriptional responses translate into changes in the physiology of the organism. In this context, the findings presented here add a new level of understanding to the efforts focused on revealing the molecular mechanisms of adaptation in the killer pathogen Mtb.

## 5 Materials and Methods

### 5.1 Mtb Strains and Growth Conditions

Mtb strain H37Rv was cultured in Middlebrook 7H9 broth supplemented with oleic albumin dextrose catalase, incubated at 37 °C on a shaker for approximately 5 days until an OD600 of 0.8. Three biological replicates were used for each experiment and time point. The sample handling and inactivation were performed as previously described in Yimer et al.^13^

### 5.2 Nitric Oxide Stress Experiment

DETA-NO was added to the broth at a final concentration of 1 mM, and Mtb cells were harvested after 30 min, 2 h, 6 h, and 20 h.

### 5.3 Proteomic Analyses

#### 5.3.1 Preparation of Cell Lysates

The Mtb cell pellets were mechanically disrupted by bead-beating with a MagNa Lyser (Roche, US), as described by Yimer et al.^13^

#### 5.3.2 In-Gel Trypsin Digestion

One hundred micrograms of gel-fractionated protein samples from Mtb cells were stained using a Colloidal Blue Staining kit (Invitrogen, CA), and each gel-lane was divided into six fractions. Each fraction was subjected to in-gel reduction, alkylation, and tryptic digestion as previously described.^47^ Proteins were reduced using 10 mM dithiotreitol (Sigma-Aldrich, Cleveland, US), alkylated with 55 mM iodoacetamide (Sigma-Aldrich, Cleveland, US), and digested with sequence grade trypsin (Promega, 1:100; w/w) overnight at 37 °C in 50 mM NH_4_HCO_3_. The in-gel digested protein samples were extracted using acetonitrile, dried in a SpeedVac concentrator (Eppendorf, concentrator 5301, US), and resuspended using 0.05% trifluoroacetic acid (Sigma-Aldrich, Cleveland, US). The extracted peptide samples were purified using C_18_ stage tips by stacking three discs from Empore. The peptides extracted from each of the six gel fractions were combined and transferred to autosampler nano-LC vials for liquid chromatography with tandem mass spectrometry (LC–MS/MS) analysis.

#### 5.3.3 Nano LC–MS/MS Analysis

Peptide characterization and quantitation were performed by nano LC–MS/MS using a Q Exactive Hybrid Quadrupole-Orbitrap Mass Spectrometer interfaced with an EASY1000-nano-electrospray ion source (Thermo Fisher Scientific, Biberach, Germany). The LC gradient was set from 2 to 30% solvent B (0.1% FA in 97% ACN) for 30 min followed by 30–75% solvent B from 30 to 35 min and 75–90% solvent B from 35 to 70 min at a flow rate of 0.3 μL/min in 50 μm × 15 cm analytical columns (PepMap RSLC, C18, 2 μm, 100 Å, Thermo Fisher Scientific). The gradient was kept at 90% solvent B from 70 to 75 min. 0.1% FA in 3% acetonitrile (ACN) (Sigma-Aldrich, Cleveland, US) and 0.1% formic acid in 97% ACN were used as solvents A and B, respectively. The mass spectrometer was operated in the data-dependent acquisition mode with automatic switching between MS and MS/MS scans.

The full MS scans were acquired at 70K resolution with an automatic gain control (AGC) target of 1 × 10^6^ ions between *m*/*z* = 300–1800 and were surveyed for a maximum injection time of 200 ms. Higher energy collision dissociation was used for peptide fragmentation at a normalized collision energy set to 28. The MS/MS scans were performed using a data-dependent top 10 method at a resolution of 17.5K with an AGC of 5 × 10^4^ ions at a maximum injection time of 100 ms and an isolation window of 2.0 *m*/*z* units. An underfill ratio of 10% and dynamic exclusion duration of 30 s were applied. For each sample group, four time points in three biological replicates were injected into the MS, resulting in a total of 24 analytical runs (two conditions, four time points, and three biological replicates).

#### 5.3.4 Peptide and Protein Identification

The MaxQuant software (version 1.6.0.16) was employed for peptide/protein identification from the raw MS data.^48^ The raw mass spectral data were searched against the Uniprot Mtb protein database containing 3993 protein sequences (Proteome ID: UP000001584, Organism ID: 83332) concatenated to reverse decoy database and protein sequences for common contaminants. Trypsin [KR].[^P] was specified as a cleavage enzyme with up to two missed cleavages. The “re-quantify” and “match between runs” options were utilized with a retention time alignment window of 3 min. Carbamidomethylation of cysteine residues was specified as a fixed modification and acetylation on protein N-terminal, conversion of N-terminal glutamine and glutamic acid to pyroglutamic acid, and oxidation of methionine were set as the variable modifications.

Both unique and razor peptides were used for the quantification of protein abundance. Peptides with a minimum length of seven amino acids and detected in at least one or more of the replicates were considered for identification. For protein identification, a minimum of two peptides, of which at least one was unique, was required per protein group. All other parameters in MaxQuant were set to default values.

### 5.4 Statistical Analysis

After filtering the data for potential contaminants and hits to the reverse database, the peptide intensities were log_2_-transformed. For peptide/protein identification, only the data having valid values in at least one sample were considered. For quantitative proteomic analysis, only the data having valid values in at least 50% of the samples were considered. The missing values were imputed from the normal distribution, and the log_2_-transformed data were normalized to Z-scores for further statistical testing. Statistical significance was determined with the multiple-sample test for the changes over the four time points and two-sample *t*-test for the changes at each time points at the *p* < 0.05 level of significance using the Perseus software (version 1.6.0.7).

### 5.5 Bioinformatics Analyses

#### 5.5.1 Gene Ontology Analysis of DA Proteins

The biological processes and molecular function for the DA proteins were identified using DAVID Bioinformatics Resources 6.7.

#### 5.5.2 Protein–Protein Interaction Network Analysis

Protein–protein interaction networks were generated via the STRING database version 10 with a high confidence threshold of 0.7 and imported into Cytoscape software (version 3.7.2) to produce the final interaction networks. Highly interconnected clusters were identified using the MCODE and ClusterOne plug-in toolkits.

#### 5.5.3 Gene Homology Searches for Mtb Rv1687c and Rv1405

Genomic sequences were downloaded from NCBI (Genbank/FASTA format). Multiple sequence alignments of *Rv1405c* and *Rv1687c* were performed with MAFFT v7.388 (scoring matrix: 200PAM/*k* = 2, gap open penalty: 1.53, offset value: 0.123), using Mtb H37Rv (NC_000962.3) as the reference genome.

### 5.6 Three-Dimensional Structure Prediction and Modeling of Mtb Rv1687c and Rv1405

The three-dimensional (3D) structure prediction for Rv1687c was retrieved from SWISS-MODEL, while PYMOL was used to generate high-quality homology models based on the *E. coli* LptB-E163Q 3D structures. The Uniprot entry of Mtb Rv1687c is https://www.uniprot.org/uniprot/O33189. The initial prediction of the tertiary structure of Mtb Rv1405c wt and G198D was performed based on the amino acid sequence alone using the web tool I-TASSER.^14,49^ Predicted structures of Rv1405c wt and G198D were superimposed with PyMOL.

## Supporting Information Available

**Supplementary Figure legends. Supplementary Figure S1.** Gene homology in *Mycobacterium tuberculosis* (Mtb) Rv1687c. Multiple sequence alignments of Rv1687c from genomic sequences (NCBI) exhibiting 70% homology to the Mtb genome, using Mtb H37Rv (NC_000962.3) as the reference genome, demonstrated that Rv1687c was highly conserved. **a.** Mtb Rv1687c homologs exhibiting 99% identity. **b.** Mtb Rv1687c homologs exhibiting 70% identity. **Supplementary Figure S2.** Gene homology in *Mycobacterium tuberculosis* (Mtb) Rv1405c. Multiple sequence alignments of Rv1405c from genomic sequences (NCBI) exhibiting 70% homology to the Mtb genome, using Mtb H37Rv (NC_000962.3) as the reference genome ,demonstrated that Rv1405c was highly conserved. **a.** Mtb Rv1405c homologs exhibiting 99% identity. **b.** Mtb Rv1405c homologs exhibiting 70% identity. **Supplementary Figure S3.** Predicted structures of Rv1405c wt and G198D were superimposed (in light blue) with the putative methyltransferase domain is highlighted in dark blue. Wildtype Gly198 is part of a short β-strand (yellow), while G198D induces a 90° rotation, resulting in a prolonged β-strand (purple). Both Asp198 and neighboring Glu272 are highlighted as sticks (a). Predicted structural changes of the putative methyltransferase domain are indicated by red arrows (b)
**Supplementary Table legends. Supplementary Table S1.** List of proteins identified in *Mycobacterium tuberculosis* (H37Rv) (n=2,911). **Supplementary Table S2.** List of proteins exclusively identified in Mycobacterium tuberculosis (H37Rv) exposed to nitric oxide stress (a) (n=186) and in control cells (b) (n=36). **Supplementary Table S3.** List of proteins with having valid values in at least 50% of the samples (n=2,490). **Supplementary Table S4.** List of differentially abundant proteins at four different time points in *Mycobacterium tuberculosis* exposed to nitric oxide stress for 30 min (a) (n=16), 2h (b) (n=79), 6h (c) (n=375) and 20h (d) (n=306)(two-sample t-test, p<0.05). **Supplementary Table S5.** The names of the DA proteins that is common in at least at two-time points. **Supplementary Table S6.** List of differentially abundant proteins (n=297) in *Mycobacterium tuberculosis* exposed to nitric oxide stress (ANOVA significant proteins, p<0.05). **Supplementary Table S7.** Gene Ontology analysis of proteins in Mtb exposed to nitrosative stress. The gene ontology analysis of DA proteins upregulated (a), and downregulated (b) at the later time points. The biological process and molecular functions enriched from DA proteins over the four time points (ANOVA significant) (c) and at each time-point combined (d). Gene ontology analysis of proteins exclusively identified in stressed *Mycobacterium tuberculosis* cells (e) (XLSX)

## Notes

The mass spectrometry proteomics data have been deposited to the ProteomeXchange consortium via the PRIDE partner repository^50^ with the data set identifier **PXD016006**.

## Abbreviations

Mtb: *Mycobacterium tuberculosis*
RNS: reactive nitrogen species
NO: nitric oxide
PTM: post-translational modification
MS: mass spectrometry
GO: gene ontology
